# Risk Factors Associated with Fatal Dengue Hemorrhagic Fever in Adults: A Case Control Study

**DOI:** 10.1155/2020/1042976

**Published:** 2020-04-30

**Authors:** Arjuna Medagama, Chamara Dalugama, Gukes Meiyalakan, Darshani Lakmali

**Affiliations:** Department of Medicine, Faculty of Medicine, University of Peradeniya, Kandy, Sri Lanka

## Abstract

**Background:**

Dengue is endemic in most parts of the tropics with a significant mortality of 1–5%. Although individual case reports and case series have been published, large-scale case controls studies are few. The objective of this study was to find clinical and laboratory predictors of mortality in dengue.

**Methods:**

Hospital case record based case control study was performed.

**Results:**

Twenty fatalities with 80 controls were analyzed. Clinical parameters of postural dizziness (OR 3.2; 95% CI 1.1–8.9), bleeding (OR 31.9; 95% CI 6.08–167.34), presence of plasma leakage (OR 64.6; 95% CI 7.45–560.5), abdominal tenderness (OR 2.24; 95% CI 0.79–6.38), and signs of cardiorespiratory instability at admission increased the risk of dying from dengue. Altered consciousness was exclusively seen in 20% of cases. Laboratory parameters of elevated CRP (OR 1.652; 95% CI 1.28–2.14), AST, or ALT > 500 IU/L (OR 52.5; 95% CI 12.52–220.1) and acute kidney injury (AKI) (OR 103.5; 95% CI 13.26–807.78) during hospital stay increased the odds of dying. Need for assisted ventilation and multiorgan dysfunction (MOD) were exclusively seen in the cases. Multivariate logistic regression revealed bleeding at admission, AKI, and elevated hepatic transaminase >500 IU/L to be independent predictors of mortality.

**Conclusions:**

This case control study revealed that mortality from dengue could be predicted using clinical parameters at admission and low cost routine laboratory investigations.

## 1. Introduction

Dengue fever is endemic in Sri Lanka and a major public health problem. The first serological confirmation of dengue in Sri Lanka was done in 1962 [1] and the first outbreak was recorded in 1965 [2].

There is an exponential increase in reported cases annually and it has become the number one killer amongst the mosquito borne infections in Sri Lanka. The Ministry of Health of Sri Lanka confirms that nearly 161,000 suspected dengue cases have been reported to the epidemiology unit during the first nine months of 2017 [3]. The last major outbreak occurred in 2009 with 35095 reported cases with case fatality rate of 1%. Although the number of new cases increased during the last seven years, the case fatality rate has decreased to less than 0.4% [3].

Death from dengue is an avoidable cause of mortality [4]. At present the case fatality rate due to dengue differs from country to country and can vary from less than 1% to much as 15% [5, 6]. Primary prevention of Dengue has limited success with vector control and other methods of primary prevention such as vaccination. Mortality reduction has therefore focused on better case management in the recent years [7, 8]. This strategy of improved clinical case management has proved successful with reduction of case fatality rate from 10–20% to less than 1% [9].

The major cause of death is dengue hemorrhagic fever (DHF)/dengue shock syndrome (DSS). Ideally the mortality from dengue should be less than 1% [4].

While this may be achieved at times when the case load is low, at times of major outbreaks the overburdening health infrastructure and service personnel may lead to increase of case fatality rates.

Sri Lanka saw the largest ever outbreak of dengue in 2017 and witnessed over 300 deaths [10]. The country and the health services struggled to cope with the current outbreak.

While most studies on dengue in the past focused on the epidemiology, diagnosis, and detection of leaking, there is hardly any data on the deaths occurring from dengue in Sri Lanka. Similarly case control studies exploring the risk factors contributing to dengue mortality are globally few.

This case control study was undertaken in this context to describe the clinicopathological features of severe dengue leading to death and to identify the risk factors contributing fatalities.

## 2. Methods

A case control study was performed using the hospital records of dengue patients admitted to Teaching Hospital Peradeniya Sri Lanka from January 2013 to September 2017. Teaching Hospital Peradeniya is a tertiary care hospital, receiving patients from primary care as well as a centre accepting complicated dengue patients for intensive care from neighboring institutions.

Institutional Ethics Review Committee (IERC) of the Faculty of Medicine, University of Peradeniya, approved the study and Institutional approval from the Director of the hospital was obtained to access the records of dengue patients.

### 2.1. Case Definition

A case was defined as a patient who was admitted with a probable diagnosis of dengue, confirmed by a positive test for the dengue-specific NS-1 antigen during the course of illness or becoming positive for dengue IgM, and died during the hospital stay following complications related to dengue illness including DHF/DSS. The controls were serologically confirmed dengue patients who developed dengue hemorrhagic fever (DHF) but making a full recovery from the illness.

## 3. Sample Size and Sampling Techniques

There were 20 adult dengue deaths on record from 2013 to September 2017 and these were selected as the cases. Patients meeting the eligibility criteria for controls were enlisted and four controls for each dengue death were randomly selected. Controls were selected from a data base of dengue patients during the period of January 2017–September 2017 admitted to Teaching Hospital Peradeniya. Total number of serologically confirmed dengue patients included 1260 among which 316 patients were diagnosed to have dengue hemorrhagic fever. Eighty patients were selected by random number from this DHF group as controls.

### 3.1. Data Collection

A trained research assistant collected data using a semistructured questionnaire. Study variables included sociodemographic characteristics, clinical details, and laboratory findings. Symptoms including the presence of fever, arthralgia, myalgia, persistent vomiting, rash, evidence of bleeding, abdominal pain, loose stools, diarrhea, altered sensorium, and reduction in urine output and important clinical signs including evidence of bleeding, rash, hypotension, tachycardia, abdominal tenderness, pallor, icterus, altered sensorium, presence of ascites, and pleural effusions were collected. Laboratory findings including full blood count, hepatic transaminases, creatinine, C-reactive protein, and dengue serology were also collected.

## 4. Data Analysis

Data entry and analysis were performed using SPSS version 20. Quantitative variables were categorized using appropriate cut-off points and medians. Student's *t*-test was used for comparing dichotomous variables of mortality associated dengue cases and those with DHF/DSS cases. For variables that were not uniformly distributed, Mann–Whitney *U* test for continuous variables was used. Logistic regression was used on data appearing to be statistically significant in the univariate analysis, to identify independent risk factors of dengue mortality. A two-tailed *P* value of <0.05 was considered statistically significant.

## 5. Results

Twenty dengue fatalities comprising 8 males and 12 females together with 80 controls comprising 46 males and 34 females were studied over the period extending from January 2013 to September 2017. There was no significant difference in the age composition between the fatalities and the controls (Table 1).

### 5.1. Disease Course

Among the fatalities, most patients were admitted to our unit on day 4 of illness (mean 3.95, range: 2–6), in most instances within two days of first medical contact. Controls were admitted on day 3 of the illness (mean 3.25, range 2–5, *P*=0.01).

All fatalities were ultimately managed in the ICU and the median day of admission to ICU was day five (mean 4.9 range: 3–7). The fatalities had a mean stay of 3.35 days in the hospital and death occurred on the seventh day of the illness (mean 6.8, range: 3–11). Ten patients had comorbidities. The cause of death in 19 of the cases was dengue hemorrhagic fever leading to multiorgan dysfunction (MOD) and massive gastrointestinal bleeding in one. The disease course of the 20 fatalities is given in Table 2.

All controls were managed in a general medical unit or HDU and were subsequently discharged home.

### 5.2. Symptoms and Signs

Fever, arthralgia, myalgia, and vomiting were common symptoms at admission in both groups. A comparison of symptoms and signs among cases and controls is given in Table 3. Postural dizziness at admission was recorded in 55% of cases and only 30% of controls (*P*=0.001) and increased the risk of dying from dengue (OR 6.4, 95% CI 1.86–22.2). Postural hypotension was also recorded in a higher percentage of cases.

Mucosal bleeding at admission was seen in 45% of fatalities and 2.5% of controls (*P* < 0.001) and increased the risk of dying from dengue 31.9 times than that of controls (OR 31.9, 95% CI 6.085–167.336).

A systolic blood pressure <90 mmHg was seen in 35% of cases and 3.7% of controls (OR 12.5, 95% CI 2.8–54.9).

Altered level of consciousness at admission was only seen among the fatalities (20%) in this study.

Features of cardiorespiratory instability (PR > 120/minute, SBP < 90 mmHg, and RR > 30/minute) at time of admission increased the odds of dying from dengue.

### 5.3. Laboratory Findings

Among the laboratory findings during hospital stay, thrombocytopenia <50,000/cumm and leukopenia were common findings in both cases and controls and were not significantly different between the fatalities and the controls (Table 4).

Elevated hepatic transaminases >500 IU/L were seen in 70% of cases and in only 6.2% of controls (*P* < 0.001, OR 52.5 95% CI 12.5–220). Similarly, an elevated CRP >2 times normal was seen in 75% of cases and 28.7% of controls (*P*=−1, OR 1.65 95% CI 1.28–2–14). A rise in creatinine and a low albumin <35 g/L were seen in greater number of fatalities compared to controls. The absolute values of selected laboratory parameters are given in Table 5.

Interestingly a platelet count <50,000/cumm was common to both cases and controls. However a platelet count <10,000/cumm was more commonly seen in the fatalities (20%) than in controls (10%, *P*=0.07 although it did not reach statistical significance.

Multiorgan dysfunction and need for assisted ventilation was exclusively seen among the fatalities.

We selected the variables with *P* < 0.1 and clinical significance in the univariate analysis for multivariate logistic regression analysis to look for the independent predictors of mortality. The selected variables were confusion, presence of mucosal bleeding, postural dizziness, hypotension, respiratory rate >30/minute, a rising creatinine, elevated CRP, and hepatic transaminases elevated >500 IU/L. The multivariate logistic regression revealed bleeding at time admission, a rising creatinine, and elevated hepatic transaminase >500 IU/L to be independent predictors of mortality.

The Hosmer–Lemeshow goodness of fit test showed a good fit in the 3 independent predictors of mortality (all *P* > 0.1).

## 6. Discussion

This case control study of dengue deaths is the first study that analyzed the clinical and laboratory features of patients dying of dengue in Sri Lanka. Compared to the previous years, an extremely high number of dengue cases and deaths were recorded during the first eight months of 2017. We believe that this analysis would provide clinicians with a greater insight into the cases that are more likely to have a complicated disease course.

Dengue deaths were primarily seen in adult female patients in the 30–59 year age group in the current study. Until 1982, Dengue mortality in South East Asia and globally was primarily considered a problem in the pediatric age group. However, the trends have changed over the last few decades and severe dengue and fatalities have become commoner among adults [11–13]. Many previous studies have reported a male preponderance in the dengue and DHF cases, but fatalities have been commoner among teenage and adult females [12–15]. However we did not observe a significant difference among the age or gender of the cases and controls.

Common clinical features of dengue were observed in the majority of cases and controls. In this study, altered level of consciousness, postural dizziness, or postural hypotension, bleeding, and evidence of plasma leakage (ascites and pleural effusions) were seen in significantly higher proportions in the cases (Table 3). Cardiorespiratory instability as evidenced by PR > 120/minute, systolic blood pressure less than 90 mmHg, and respiratory rate greater than 30/minute were also significantly higher among the cases.

Hemorrhagic manifestations were observed in 45% of cases at admission. Other studies have also reported similar findings. Karunakaran et al. [8] reported 35% of fatalities to develop bleeding manifestations while Assir et al. reported some form of bleeding manifestation in more than 50% of fatalities [16]. Interestingly logistic regression also identified bleeding at time of admission to be an independent predictor of death in the current study.

Altered sensorium in dengue infection is indicative of CNS involvement and associated with poor prognosis [17] and higher mortality [8]. Coma at presentation [18] is associated with higher mortality and multiorgan involvement [19, 20]. Twenty percent of our cases and none of the controls had altered sensorium at time of admission. Encephalopathy may be due to the presence of liver failure [21], the neurotropic effect of the virus [19, 22], multiorgan involvement, and sepsis. Two fatalities of the current study had features of severe liver involvement. Our findings are in alignment with those of Woon et al. reporting CNS involvement in 25% of their fatalities [23] and in contrast to those of Karunakaran reporting CNS involvement in 80% of fatalities [8]. As the timing of assessment for CNS involvement differs among studies, more robust data is required to draw definitive conclusions.

Although the cause of altered sensorium in our patients could be multifactorial, the finding of altered level consciousness should alert the clinician to a possible adverse outcome.

All fatalities had at least 1 warning sign at the time of admission (Table 3), indicating that they were at an advanced stage of the disease. Thirty-five percent of our cases were already in shock requiring immediate fluid resuscitation. However, Woon et al. from Malaysia reported that 21.9% of deaths did not have any warning sign at time of admission [23]. Other studies reporting on the presence of warning signs have not specifically reported the presence of these signs at admission. Mallhi et al. in Malaysia [24] and Guzman et al. from Cuba [5] reported all fatalities to have at least 1 warning sign. Contrary to these findings Ong et al. reported the presence of warning signs in 50% of dengue deaths [25]. Presence of at least one warning sign has been reported in 58–78% of fatalities in other studies [23]. However, it is not clear whether these were recorded at time of admission of later and thereby limiting their interpretation. Based on our findings and those of Woon et al. [23], the presence of warning signs at admission should alert the attending medical team to a possible adverse outcome.

Previous studies have reported persistent vomiting, abdominal pain, and hepatomegaly [23, 26] to be more frequent in fatalities. However, in our study these symptoms and signs seemed to be common to both cases and controls and the difference did not reach statistical significance.

In this case control study, bleeding, postural dizziness, and postural hypotension and plasma leakage were statistically higher among the deaths at admission compared to others with DHF/DSS and should alert the physician to adverse outcomes.

Some studies have demonstrated that early presentation to medical care may improve and avoid fatal outcomes in dengue [12, 15]. In our study, those dying of dengue were admitted to hospital one day later than the survivors (cases on day 4 or later and controls on day 3 or earlier, *P*=0.016). This was then followed by a rapid deterioration and death occurring within 2-3 days. Similarly Mallhi et al. found a significant difference in the duration of illness prior to admission between the fatalities and controls [24]. Woon et al. also found that deaths were more common in patients admitted after the 4^th^ or 5^th^ day of the illness [23]. These observations are consistent with earlier studies done on dengue deaths where later presentation to hospital was found to be a likely cause for increased mortality [5, 12, 25]. These findings highlight the importance of encouraging those with fever to seek early medical care and the need for early, institutionalized care in dengue.

During the course of the hospital stay, a rise in CRP greater than 2-fold, elevated creatinine and elevation of hepatic transaminases >500 IU/ml were seen in significantly higher proportions in the cases, while MOD and need for ventilatory support were exclusively seen in the cases.

We observed 90% of our fatalities to develop AKI. AKI is a well-recognized association with dengue mortality and has been previously reported. Khalil et al. and Mallhi et al. reported AKI in all fatal cases [24, 27]. In another case series, Lee et al. reported AKI in 80% of patients dying of dengue [28]. Our findings are therefore largely in alignment with the previous findings.

In the current case control study, mild elevation of ALT and AST was common to both cases and controls and did not reach statistical significance. However, 70% of cases and only 6% of controls had elevated hepatic transaminases >500/L (*P* < 0.001). Elevated transaminases are indicative of hepatitis. Dengue virus is hepatotrophic and known to cause hepatitis of varying severity and sometimes results in massive hepatic necrosis [29]. Prolonged hypotension and shock in cases of DHF and DSS could be another cause leading hepatic damage. Transaminases in excess of 300 mg/dl have been reported to be associated with dengue mortality [30]. Sam et al. also reported elevated transaminases in excess of 1000 IU/L in most of the dengue fatalities [12] and Woon et al. demonstrated severe liver involvement in over 60% of their fatalities [23]. Fernando et al. found elevation of transaminases in exceeding 1000 IU/L in patients with severe dengue in Sri Lanka [31]. These findings are largely in keeping with our results and rising transaminases in excess of 500 IU/L should be considered a predictor of complicated dengue and mortality.

Fifty percent of fatalities in this study had elevated CRP on admission and most (75%) progressed to have high CRP within the hospital stay. In contrast, an elevation in CRP was seen in only 28% of controls (*P* < 0.001). In absolute terms, fatalities had a mean CRP of 107.9 in contrast to 19.5 of controls (*P* < 0.001). CRP is an acute phase protein synthesized in the liver, which is elevated in infections, inflammation, burns, trauma, and malignancy. It is also often used to distinguish viral infections from bacterial ones [32]. The reason for elevation of CRP in complicated dengue patients could be manifold.

In the current study, bacterial cultures were not available in all patients thus making it difficult to ascertain the exact rise for the CRP elevation. Secondary infections and hepatic necrosis may have contributed with the significantly elevated CRP values in the fatalities compared to controls in this study. In previous studies, secondary bacteraemia leading to elevation of CRP and contributing to mortality has been reported [25, 33, 34]. However, elevation of CRP is recognized as a predictor of mortality in this study.

In the current study the platelet counts were similar between the fatalities and the controls. Most patients (90% in cases and 91% in controls) had platelet counts less than 50,000/cumm. A count less than 10,000/cumm was seen in higher percentages of cases (20%) than controls (10%). Although an attendant low platelet count is seen in most dengue infections, a platelet count less than 50,000 has been shown to increase mortality by sixfold in a study done by Chua et al. [35]. Other studies have also established platelet counts less than 50,000 to be associated with higher mortality [8, 12]. In the Study by Karunakaran et al., the controls were noncomplicated dengue patients with dengue fever, while that of Sam et al. was a retrospective case analysis. The almost identical low percentages of thrombocytopenia witnessed among cases and controls in our study can be accounted by the use of patients with DHF/DSS as controls as they will have lower platelet counts. Therefore although a platelet count <50,000 may be predictor of death in patients with dengue fever, it fails to recognize those at risk of death among a cohort of DHF/DSS.

We witnessed a significantly lower albumin value among cases compared to controls. This is explained probably by the greater plasma leakage and the liver dysfunction witnessed in these patients. Meta-analysis performed by Huy et al. showed that hypoalbuminaemia was a consistent finding in [36] DSS across 13 studies. However, ours is the first study demonstrate hypoalbuminaemia to occur with greater frequency among fatalities.

## 7. Strengths and Limitations

This study is one of the first case control studies exploring the risk factors of dengue mortality. Although DHF/DSS is not uncommon, patients dying from dengue are rare. Previous studies have utilized smaller samples. In this study as we have used patients with DHF as controls, the risk factors that discriminate death from DHF are highlighted. As limitations we acknowledge the limitations inherent to retrospective case record based studies. We also acknowledge that this is a single centre study.

## 8. Conclusions

This study confirmed the presence of bleeding manifestations (*P* < 0.001), altered level of consciousness (*P* < 0.001), and evidence of plasma leakage at time of admission (*P* < 0.001) as important clinical predictors of mortality. Similarly elevated transaminases >500 IU/L (*P* < 0.001), elevated creatinine (*P* < 0.001), and CRP (*P*=0.001) were laboratory predictors of death.

## Figures and Tables

**Table 1:** Age, gender, selected admission, and management details.

		Controls		Cases		*P* value
1	Gender	Male *n* (%)	46 (57.5%)	Male *n* (%)	8 (40%)	0.16
		Female *n* (%)	34 (42.5%)	Female *n* (%)	12 (60%)	

2	Age (yrs)	Mean (SD)	32.8 (13.65)	Mean (SD)	37.35 (16.5)	0.20
		Range	13–56	Range	13–75	

3	Admitted on day	Mean (SD)	3.25 (1.17)	Mean (SD)	3.95 (0.99)	0.016
		Range	2–5	Range	2–6	

4	Place of management	Ward/HDU	80	Ward/HDU	0	
		ICU	0	ICU	20	

**Table 2:** Clinical symptoms and selected physical signs of fatalities on admission.

Clinical features on admission																										
	Patients directly admitted to THP (*n* = 9)											Patients transferred (*n* = 11)													Overall	
Serial no.	1	2	3	4	5	6	7	8	9			10	11	12	13	14	15	16	17	18	19	20				
Patient no.	1	4	5	10	12	15	16	17	19	(*N*)	%	2	3	6	7	8	9	11	13	14	18	20	Frq.	%	Frq.	%
Headache		+					+			2	22.2								+				1	9	3	15
Altered consciousness	−		−	−	−	−	−	+	−	1	11.1	−			+	+	−	−	+	−	−	−	3	27.3	4	20
Arthralgia/myalgia	+	+	+				+			4	44.4			+					+				2	18.2	6	30
Fever	+		+	−	−	−				2	22.2	+	+	−	+	−		+	−	+	+	−	6	54.5	8	40
Nausea/vomiting	+	+		+	−	−	+	+	+	6	66.7	−			−			+		+		+	3	27.3	9	45
Abdominal pain			−	+	+	−		−		2	22.2	−			+			+	−	+		+	4	36.4	6	30
Postural dizziness		+	−	+	+	+	+	+	+	7	77.8	−		+	−			+		+	+	−	4	36.4	11	55
Bleeding	−	+	−	−	+	−	−	−	−	2	22.2	+	+	+	−	+	+	+	+	−	−	−	7	63.6	9	45
Diarrhea				+				−	−	1	11.1								−	+	−	−	1	9	2	10
RH pain									+	1	11.1												0	0	1	5
Presence of at least 1 warning sign	+	+	−	+	+	+	+	+	+	8	88.9	+	+	+	+	+	+	+	+	+	+	+	11	100	19	95
Abdominal tenderness	−	+		+	+	+	+	+	−	6	66.7	−	+	−	+		−	+	−	+	−	+	5	45.5	11	55
Confusion	−		−	−	−	−	−	+	−	1	11.1	−			−	+	+	−	+	−	−	−	3	27.3	4	20
Pleural effusion	−	−	−	−	+	+	−	−	−	2	22.2	+	−	+	+	−	+	+	+	+	−	−	7	63.6	9	45
Ascites	−	−	−	−	−	−	+	−	−	1	11.1	+	−	+	+	+	−	−	−	+	−	−	5	45.5	6	30
Hepatomegaly	−	+	−	−	−	−	−	−	−	1	11.1	−	−	−	−	−	−	−	−	−	−	−	0	0	1	5
Oliguria	−	−	−	−	−	−	+	+		2	22.2	+	+	−	+	+	+	−	−	+	−		6	54.5	8	40
RR > 30/minute	−	−	−	−	−	−	−	−		0	0	−	−	+	−	−	+	−	−	+	+	−	4	36.4	4	20
PR > 120/minute	−	−	−	−	−	−	−	−	−	0	0	+	+	+	+	−	+	−	+	+	+	−	8	72.7	8	40
SBP < 90 mmHg	−	+	−	−	−	+	+	−	−	3	33.3	−	+	+	−	+	−	−	−	+	−	−	4	63.6	7	35
Dehydration	−	+	+					+		3	33.3											+	1	9	4	20
Shock	−						+			1	11.1	+	+	+		+	+		+				6	54.5	7	35

THP: Teaching Hospital Peradeniya, RH: right hypochondriac, RR: respiratory rate, PR: pulse rate, SBP: systolic blood pressure.

**Table 3:** Selected signs and symptoms on admission.

	Symptom	Controls *n*, (%)	Cases *n*, (%)	*P* value	Odds ratio (95% CI)
1	Age >40 years	20 (25)	7 (35)	0.36	1.6 (0.5–4.6)
2	Headache	55 (68.7)	3 (15)	0.01	0.82 (0.02–0.31)
3	Arthralgia/myalgia	68 (85)	6 (30)	0.350	1.08 (1.02–1.16)
4	Fever	55 (68.7)	8 (40)	0.219	0.49 (0.16–1.53)
5	Persistent vomiting	20 (25)	6 (30)	0.60	1.27 (0.42–3.7)
6	Abdominal pain	26 (32.5)	6 (30)	0.151	0.89 (0.3–2.58)
7	Postural dizziness	24 (30)	11 (55)	0.02	3.2 (1.1–8.9)
8	Bleeding	2 (2.5)	9 (45)	0.0001	31.9 (6.08–167.34)
9	Diarrhea	14 (17.5)	2 (10)	0.468	1.8 (0.33–10.72)
10	Right upper quadrant pain	3 (3.7)	1 (5)	0.0001	1.3 (0.76–2.35)
11	Altered consciousness	0	4 (20)	0.0001	

Signs					
12	Abdominal tenderness	33 (41.2)	11 (55)	0.126	2.24 (0.79–6.38)
13	Mucosal bleeding	1 (1.2)	9 (45)	0.0001	64.6 (7.45–560.5)
14	Pleural effusion	1 (1.2)	9 (45)	0.0001	64.6 (7.45–560.5)
15	Ascites	1 (1.2)	6 (30)	0.0001	33.8 (3.7–303)
16	Hepatomegaly	3 (2)	1 (5)	0.044	0.192 (0.13–0.29)
17	Postural drop in BP	24 (30)	10 (50)	0.007	4.583 (1.14–14.85)
18	RR > 30	3 (3.7)	14 (70)	0.0001	59 (13.3–264.6)
19	PR > 120	1 (1.2)	8 (40)	0.0001	48 (5.49–419)
20	SBP < 90 mmHg	3 (3.7)	7 (35)	0.0001	12.564 (2.87–54.99)

RR: respiratory rate, PR: pulse rate, BP: blood pressure, SBP: systolic blood pressure.

**Table 4:** Selected clinical and laboratory findings during hospital stay.

	Parameters	Controls *n*, (%)	Cases *n*, (%)	*P* value	Odds ratio (95% CI)
1	CRP rise	23 (28.7)	15 (75)	0.001	1.652 (1.28–2.14)
2	Liver enzyme > 2 times	73 (91.2)	17 (85)	0.655	1.630 (0.19–14.15)
3	Liver enzyme > 500	5 (6.2)	14 (70)	0.0001	52.5 (12.52–220.1)
4	Platelet count < 10	8 (10)	5 (20)	0.074	3.0 (0.86–10.45)
5	Platelet count < 50	73 (91.2)	18 (90)	0.07	0.863 (0.16–4.51)
6	Albumin < 3.5 (g/L)	17 (21.2)	19 (95)	0.001	1.82 (1.32–2.51)
7	Creatinine rise	2 (2.4)	18 (90)	0.0001	103.5 (13.26–807.78)
8	Evidence of myocarditis	1 (1)	5 (25)	0.0001	0.158 (0.10–0.25)
9	MOD	0	19 (95)	0.0001	
10	Ventilation	0	20 (100)	0.0001	

CRP: C-reactive protein, MOD: multiorgan dysfunction.

**Table 5:** Absolute values of selected laboratory parameters during hospital stay.

	Parameter	Cases (SD)	Controls (SD)	*P* value
1	Creatinine (Umol/L)	293.58 (133.0)	75.08 (21.2)	0.0001
2	AST (IU/L)	7,145.64 (7,614.4)	198.20 (179.3)	0.0001
3	ALT (IU/L)	2,760.16 (2,761.4)	133.88 (122.2)	0.0001
4	CRP (mg/L)	107.92 (109.1)	19.54 (23.3)	0.0001
5	Platelet count (^*∗*^1,000/cumm)	25.60 (33.2)	28.24 (19.1)	0.640
6	Albumin (g/L)	21.9 (5.5)	36.1 (9.2)	0.0001

## Data Availability

The data sets used and/or analyzed during the current study are available from the corresponding author on reasonable request.

## Abbreviations

DHF: Dengue hemorrhagic fever
DSS: Dengue shock syndrome
AKI: Acute kidney injury
HDU: High dependency unit
CRP: C-reactive protein
ALT: Alanine aminotransferase
AST: Aspartate aminotransferase
ICU: Intensive care unit
MOD: Multiorgan dysfunction.
